# Diastolic function and cardiovascular events in patients with preserved left ventricular ejection fraction. Improving risk stratification with left atrial strain

**DOI:** 10.3389/fcvm.2025.1565052

**Published:** 2025-07-09

**Authors:** Ibon Rodriguez-Sanchez, Inaki Villanueva-Benito, Urko Agirre, Jose Juan Onaindia, Alazne Urkullu, Angela Cacicedo, Alberto Ullate, Idoia Bravo, Josu Florido, Alberto Salcedo, Sonia Velasco

**Affiliations:** ^1^ Department of Cardiology, Galdakao University Hospital, Biobizkaia Health Research Institute, Osakidetza, Spain; ^2^ Department of Cardiology, Donostia University Hospital, Biodonostia Health Research Institute, Osakidetza, Spain; ^3^ Research Unit, Red de Investigación en Servicios de Salud en Enfermedades Crónicas (REDISSEC), Galdakao, Spain

**Keywords:** diastolic function, left atrial strain, risk stratification, preserved LVEF, prognosis, heart failure, atrial fibrillation, ischemic stroke

## Abstract

**Background:**

A limited number of studies have examined the prognostic significance of diastolic function in patients with preserved left ventricular ejection fraction (LVEF) in a general population referred for transthoracic echocardiography. Our aim was to assess the association between diastolic function and a combined event in which the left atrium plays a pivotal role, including heart failure (HF), atrial fibrillation (AF) and ischemic stroke. The study sought to determine the incremental value of left atrial reservoir strain (LARS) in risk stratification.

**Methods:**

We performed a retrospective analysis of 364 patients with preserved LVEF and sinus rhythm referred for transthoracic echocardiography and categorized them into four groups based on their diastolic function status according to the 2016 ASE/EACVI guidelines: normal diastolic function (NDF), indeterminate diastolic function and diastolic dysfunction with indeterminate filling pressure (IDT), grade 1 diastolic dysfunction (DD1), and diastolic dysfunction with elevated filling pressure (DD-EFP). The primary endpoint was a composite of HF, AF or ischemic stroke. LARS was measured by 2D speckle tracking. Clinical parameters, comorbidities and specific cardiac diseases were also assessed. Secondary endpoint was all-cause mortality.

**Results:**

The mean follow-up period was 2.4 years. IDT and DD-EFP diastolic function status were independently associated with the combined event. The incorporation of LARS enhanced risk stratification, particularly in IDT patients, with a cutoff of ≤24% identifying a high-risk population. Patients classified as high risk, defined as those with DD-EFP and IDT with LARS ≤ 24%, exhibited a notable event rate of 34% and 46%, respectively. Diastolic function and LARS were not independently associated with all-cause mortality.

**Conclusions:**

In patients with preserved LVEF and sinus rhythm, diastolic function is strongly and independently associated with the combined event of HF, AF, or ischemic stroke. LARS provides a valuable tool for improving risk stratification in this population. Patients at high risk (DD-EFP and IDT with LARS ≤ 24%) demonstrated a significant event rate, underscoring the necessity for preventive measures. Diastolic function and LARS were not independently associated with all-cause mortality. Further studies are required to confirm these findings and validate the proposed approach.

## Graphical Abstract

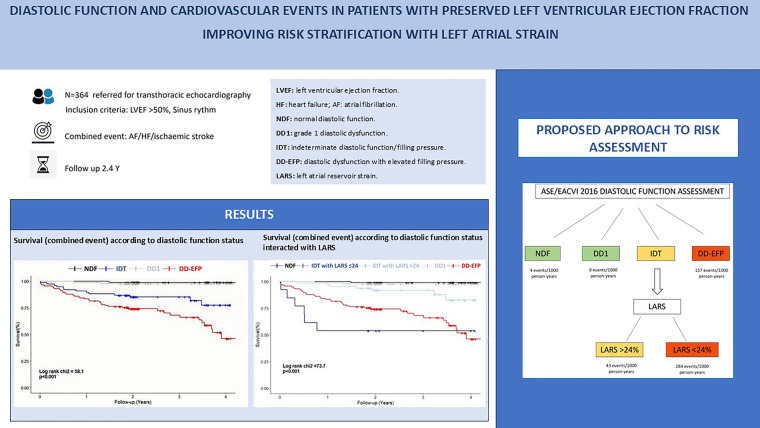

## 1 Introduction

Assessing diastolic function is crucial, especially in patients with preserved left ventricular ejection fraction (LVEF). Diastolic function plays a major role in the occurrence of events such as atrial fibrillation (AF), heart failure (HF), and ischemic stroke (1). Although this has been investigated in various heart diseases, the association of diastolic function with the occurrence of such events irrespective of specific cardiopathy, using the 2016 American Society of Echocardiography/European Association of Cardiovascular Imaging (ASE/EACVI) recommendations, is poorly established (2, 3).

While current guidelines for assessing diastolic function (4) represent a major advancement, they are complex and involve multiple parameters. A significant proportion of patients is classified as having indeterminate diastolic function or filling pressure after applying their algorithms (2). Recently, the left atrial reservoir strain (LARS) with a cutoff value of <18% has been proposed to identify patients with diastolic dysfunction and elevated filling pressure when one of the three main criteria is unavailable (E/e’ ratio, tricuspid regurgitation velocity, and left atrial volume index) (5). However, this LARS cutoff value exhibits greater accuracy in patients with reduced LVEF than in those with preserved LVEF (6).

Mortality or hospitalization for HF is the primary outcome in most studies. All-cause mortality in patients with preserved LVEF is often determined by non-cardiac causes (comorbidities). We aimed to focus on a potentially preventable cardiac event, but adjust for and account for extracardiac comorbidities. In addition to hospitalization for HF, AF and ischemic stroke are clinical events with a major impact on morbidity and quality of life that can occur in patients with preserved ejection fraction, especially in the presence of atrial myopathy.

Our study sought to (1) evaluate the association of diastolic function with the occurrence of a combined event of HF, AF or ischemic stroke in a general cardiologic population of patients with preserved LVEF and sinus rhythm referred for echocardiography, independently of specific heart disease and (2) determine the added value of LARS in the risk stratification of these patients, given the substantial role of left atrial disease in this combined event and (3) evaluate the association of diastolic function and LARS with all-cause mortality in our population.

## 2 Materials and methods

This observational, retrospective, single-centre study included 364 consecutive patients referred for transthoracic echocardiography by their treating physician during 2018–2020 period. Both outpatients and inpatients were included, concluding recruitment when each diastolic function group reached 80 patients. The determination of this sample size was arbitrarily made based on previously published studies (6–9). Due to the challenges associated with recruiting patients exhibiting grade 3 diastolic dysfunction while preserving LVEF and sinus rhythm, those with elevated filling pressure (grade 2 and grade 3 diastolic dysfunction) were pooled for analysis, constituting the largest group. In this way, we achieved that all diastolic groups were represented in the studied cohort.

Patients with an LVEF of >50% and stable sinus rhythm were considered eligible for the study. Meanwhile, patients with non-sinus rhythm detected on echocardiography, moderate-to-severe or severe mitral regurgitation, mitral stenosis, mitral valve prosthesis, pacemaker implanted, or poor-quality images preventing LARS analysis were excluded. The patient selection process is depicted in Supplementary Figure S1. The study was approved by the Institutional Ethics Committees, and the requirement for obtaining informed consent was waived owing to the retrospective nature of the study.

### 2.1 Echocardiographic measurements

A conventional echocardiographic examination was performed as prescribed by the treating physician at the EACVI-accredited echocardiography laboratory at our institution.

Diastolic function was assessed according to the 2016 ASE/EACVI guidelines. Diastolic dysfunction was defined according to the A algorithm for the assessment of LV diastolic function with normal EF, i.e., >50% positive of these 4 parameters: 1-Average E/e’ >14; 2-Septal e’ velocity <7 cm/s or lateral e’ velocity <10 cm/s; 3-TR velocity > 2.8 m/s; 4-LV volume index >34 ml/m^2^. If diastolic dysfunction was diagnosed, algorithm B was used to determine normal (grade 1 diastolic dysfunction) or elevated left atrial pressure (grade 2 and 3 diastolic dysfunction) (4). Patients were classified according to their diastolic function status and distributed into the following groups for analysis: normal diastolic function (NDF), grade 1 diastolic dysfunction (DD1), diastolic dysfunction with elevated filling pressure (DD-EFP: patients with grade 2 and 3 diastolic dysfunction), and indeterminate group (IDT: patients with indeterminate diastolic function and patients with diastolic dysfunction and indeterminate filling pressure). In addition, other common echocardiographic parameters were assessed (Table 1). Echocardiographic measurements were performed blinded to all clinical events.

**Table 1:** Baseline characteristics. Demographics, clinical history and echocardiographic parameters.

Characteristic	NDF (*N* = 88)	IDT (*N* = 76)	DD1 (*N* = 86)	DD-EFP (*N* = 114)	Total (*N* = 364)	*P* value
Demographics						
Sex, female	39 (44%)	32 (42%)	45 (52%)	79 (69%)	195 (53%)	<0.001
Age, y	54 ± 14	68 ± 11	69 ± 10	76 ± 10	68 ± 14	<0.001
Body mass index, (kg/m^2^)						0.001
<25	43 (49%)	17 (23%)	24 (28%)	30 (26%)	114 (31%)	
25–30	30 (34%)	35 (46%)	30 (35%)	41 (36%)	136 (38%)	
≥30	15 (17%)	23 (31%)	32 (37%)	43 (38%)	113 (31%)	
Clinical history						
Hypertension	34 (39%)	48 (63%)	65 (76%)	93 (82%)	240 (66%)	<0.001
Diabetes	12 (14%)	14 (18%)	25 (29%)	41 (36%)	92 (25%)	<0.001
Hyperlipemia	36 (41%)	51 (67%)	48 (56%)	78 (68%)	213 (59%)	<0.001
History of heart failure	0 (0)	5 (7%)	2 (2%)	19 (17%)	26 (7%)	<0.001
History of atrial fibrillation	2 (2%)	11 (14%)	10 (12%)	23 (20%)	46 (13%)	<0.001
History of stroke	3 (3%)	8 (11%)	9 (10%)	14 (12%)	34 (9%)	0.17
COPD/asthma	14 (16%)	15 (20%)	9 (10%)	19 (17%)	57 (16%)	0.42
Serum creatinine^a^	0.9 (0.7, 2.7)	0.9 (0.7, 1.3)	0.9 (0.6, 1.1)	0.9 (0.8, 1.0)	0.9 (0.7, 1.1)	0.03
Heart disease						<0.001
No heart disease	58 (71%)	14 (21%)	9 (12%)	5 (5%)	86 (26%)	
Ischemic heart disease	5 (6%)	12 (18%)	9 (12%)	10 (10%)	36 (11%)	
Severe aortic stenosis	0 (0%)	5 (7%)	4 (5%)	14 (13%)	23 (7%)	
Hypertensive heart disease	10 (12%)	22 (32%)	21 (27%)	50 (48%)	103 (31%)	
Valvular heart disease	6 (7%)	13 (19%)	23 (29%)	17 (16%)	59 (18%)	
Amyloidosis	0 (0%)	1 (1%)	1 (1%)	2 (2%)	4 (1%)	
Others	3 (4%)	1 (1%)	11 (14%)	6 (6%)	21 (6%)	
NYHA class						<0.001
1	72 (81.82)	58 (76.32)	60 (70.59)	52 (47.27)	242 (67.41)	
2	16 (18.18)	17 (22.37)	23 (27.06)	47 (42.73)	103 (28.69)	
3	0 (0)	0 (0)	2 (2.35)	9 (8.18)	11 (3.06)	
4	0 (0)	1 (1.32)	0 (0)	2 (1.82)	3 (0.84)	
Echocardiographic parameters						
LVEF (%)	64 ± 7	65 ± 6	64 ± 8	65 ± 7	64 ± 7	0.53
E/A ratio	1.1 ± 0.4	0.9 ± 0.3	0.8 ± 0.3	1.1 ± 0.5	0.9 ± 0.4	<0.001
e' average (cm/s)	9.5 ± 2.7	7.3 ± 1.9	7.3 ± 5.3	6.2 ± 4.1	7.5 ± 3.9	<0.001
LAVi (ml/m^2^)	26 ± 9	37 ± 7	40 ± 10	49 ± 12	38 ± 13	<0.001
E/e' average	8.2 ± 2.4	10.2 ± 2.6	10.3 ± 2.2	17.5 ± 7.0)	12.0 ± 5.8	<0.001
TR velocity (cm/s)	237 ± 26	243 ± 32	242 ± 21	291 ± 39	259 ± 39	<0.001
LV mass (g/m^2^)	98 ± 24	111 ± 25	118 ± 29	132 ± 38	116 ± 33	<0.001
RWT	0.39 ± 0.07	0.43 ± 0.10	0.45 ± 0.11	0.47 ± 0.11	0.44 ± 0.10	<0.001
LARS	39 ± 9	31 ± 8	32 ± 8	23 ± 8	31 ± 10	<0.001
LV global longitudinal strain (%)	21.9 ± 1.9	22.2 ± 2.5	21.4 ± 3.6	19.3 ± 2.3	20.8 ± 2.8	<0.001

Demographics, clinical history and echocardiographic parameters.

Data are shown as frequency (column percentage) and mean ± standard deviation. NDF, normal diastolic function; DD1, grade 1 diastolic dysfunction; IDT, indeterminate diastolic function/filling pressure; DD-EFP, diastolic dysfunction with elevated filling pressure; COPD, chronic obstructive pulmonary disease; NYHA, New York Heart Association; LVEF, left ventricular ejection fraction; LAVi, left atrial volume index; TR, tricuspid regurgitation; RWT, relative wall thickness; LARS, left atrial reservoir strain; LV, left ventricular.

^a^ Results displayed as median (*P*_25_ − *P*_75_).

### 2.2 Speckle-tracking measurements

LARS was measured through two-dimensional speckle-tracking analyses in the apical 4- and 2-chamber views, and a mean value was obtained. The images were obtained in apical 4 and 2-chamber view adjusted to visualize the entire left atrium, avoiding fore-shortening, with a minimum frame rate of 50 fps. The software used was PHILIPS QLAB VERSION 10, in its version for left ventricle analysis, but focusing on the left atrium, with R-R gating. The operator manually marked the septal and lateral bases (4-chamber view) or the inferior and anterior bases (2-chamber view) and the roof of the left atrium to semi-automatically initiate the ROIs. Pulmonary veins and left atrial appendage were not included. The ROIs were manually corrected in cases where tracking was inaccurate due to poor image quality. LARS was assessed by an expert operator who was blinded to all clinical events. Global longitudinal strain of the left ventricle is reported as an absolute value.

### 2.3 Clinical data and events

Sociodemographic and anthropometric variables were assessed, along with the cardiovascular risk factors, comorbidities, and analytical parameters such as serum creatinine levels. Data on each patient's heart disease were collected to analyse the possible confounding effect (Table 1).

The primary endpoint was defined as a combined event of HF, AF, or ischemic stroke, whichever occurred first from the date of the echocardiographic examination. Data on events were retrieved from the electronic medical records, ensuring blindness to the echocardiographic data. HF was defined as hospital admission for HF, accompanied by elevated N-terminal pro b-type natriuretic peptide (>400 pg/ml). AF was defined as clinical AF (evaluated on a 12-lead ECG or Holter ECG at a duration >30 s). Ischemic stroke was defined as ischemic stroke leading to hospital admission, documented in the neurology discharge report. Transient ischemic attacks were not included.

### 2.4 Statistical analysis

The study presented a detailed description of the sociodemographic, clinical, and echocardiographic characteristics of eligible patients based on their diastolic function status. The measures of central tendency (means and medians) and the measures of variability (standard deviations and interquartile range) were calculated for continuous variables. Frequencies and percentages were computed for categorical variables. To evaluate the relationship between the characteristics described above and the type of diastolic function, various statistical tests were employed. The Kruskal–Wallis non-parametric test was utilised for comparing continuous data, while the chi-square test was employed for comparing categorical variables.

The variable of interest in our study was the occurrence or non-occurrence of heart failure, atrial fibrillation, and stroke. The primary goal was to model the initial instance of any of these events. The time to the event was calculated by subtracting the date of the first study evaluation from the date of the first occurrence of these events, irrespective of their nature. To evaluate the influence of both diastolic function and the LARS index on the prognosis of outcome development, unadjusted and adjusted Cox regression models were constructed. In the adjusted or multivariate models, independent variables were those whose *p*-value was less than 0.20 in the unadjusted analysis (10). The hazard ratios (HRs) and their corresponding 95% confidence intervals were used to assess the outcomes from the Cox regression models. The aforementioned methodology was also employed in evaluating the remaining variables obtained in the study. Kaplan–Meier survival curves were depicted and the log-rank test was applied to study the statistical significant differences among the assessed groups. Furthermore, our objective was to establish the optimal threshold of the LARS index that can effectively discriminate events from non-events, while achieving a more equitable trade-off between sensitivity and specificity. To achieve this objective, the screening parameters, including sensitivity, specificity, positive and negative predictive values, and accuracy, were calculated.

Additionally, three multivariate Cox regression models were conducted. The initial model incorporated the assessment of diastolic function (Model 1). The next models included the assessment of diastolic function and the categorization of the LARS index, treating the latter variable as both a main effect (Model 3) and interacting with diastolic function (Model 2). In Model 2, we hypothesized that the prognosis in the IDT patient group was different according to the established LARS groups. Thus, a five-level diastolic-LARS combined exposure variable was created: NDF, DD1, IDT patients with LARS < 24%, IDT patients with LARS > 24% and DD-EFP. All models were adjusted for gender and patients’ age, body mass index, hyperlipidaemia, presence of chronic obstructive pulmonary disease/asthma, echocardiographic parameters including LVEF, and heart disease. Furthermore, a likelihood-ratio test was conducted to compare the goodness of fit among the developed models. Besides, the net reclassification index (NRI) was computed. The boostrap resampling method was utilised to assess the stability of the Harrell's C statistic across 2,000 repeats. A value >0.8 was considered a good discrimination ability.

The statistical approaches utilised in this study were designed using the SAS System and R v4.2 statistical software. A *p*-value of less than 0.05 was considered significant.

## 3 Results

### 3.1 Study population

Of 436 patients who met the inclusion criteria, 72 were excluded due to poor-quality image or unsuitability for 2016 ASE/EACVI diastolic function assessment algorithms. The patient selection process is depicted in Supplementary Figure S1.

### 3.2 Baseline characteristics

A total of 364 patients (53% women) were included, with a mean age of 68 years (±14) and a Simpson's LVEF of 64% (±7). The baseline characteristics of the study population are summarised in Table 1. The patients were divided into four groups based on their diastolic function: 88 patients with NDF, 86 patients with DD1, 114 patients with DD-EFP (DD2: 100 patients and DD3: 14 patients; data on patients with grade 2 and 3 diastolic function are included in the Supplementary Material S3, S4), and 76 patients with IDT. Our study included 21% of patients in the IDT group, which is consistent with the proportion of patients observed in other studies (2). The majority of patients with NDF had no significant heart disease (70%), unlike those with diastolic dysfunction. Patients who demonstrated deteriorating diastolic function were older, had more comorbidities, had greater LV hypertrophy and LV concentric remodelling, and had lower LARS values. LARS values also decreased with age and were lower in women, patients with cardiovascular risk factors, and patients with a medical history of HF, AF, or ischemic stroke (Table 1).

### 3.3 Primary endpoint: combined event

After a mean follow-up of 2.4 (±1.0) years, 58 events were recorded in 55 patients (15% of the sample): 20 events of HF, 28 events of AF, and 10 events of ischemic stroke (with three patients experiencing two events on the same date: AF and HF, and AF and ischemic stroke). Patients who had events were older, with more advanced heart disease, and with a medical history of AF, bronchopathy, and dyslipidaemia. Based on the echocardiographic data, patients who experienced events had worse diastolic function parameters, LARS, lower LVEF and LV global longitudinal strain, and greater LV hypertrophy and LV concentric remodelling (Tables 2, 3).

**Table 2:** Univariate survival analysis for the presence of the combined event (heart failure, atrial fibrillation or ischemic stroke). Demographics, clinical and standard echocardiographic parameters.

Characteristic	No event (*n* = 309)	Event (*n* = 55)	HR (95% CI)	*P* value
Demographics				
Sex, female, *n* (%)	163 (53%)	32 (58%)	1.09 (0.64, 1.87)	0.75
Age, y	66 ± 14	75 ± 9	1.06 (1.03, 1.09)	<0.001
Body mass index, (kg/m^2^)				
<25	99 (32%)	15 (27%)	Reference	
25–30	111 (36%)	25 (45%)	1.49 (0.78, 2.82)	0.23
≥30	98 (32%)	15 (27%)	0.95 (0.6, 1.94)	0.88
Clinical history				
Hypertension	197 (64%)	43 (78%)	1.85 (0.98, 3.50)	0.06
Diabetes	75 (24%)	17 (31%)	1.28 (0.72, 2.27)	0.40
Hyperlipidemia	171 (55%)	42 (76%)	2.45 (1.32, 4.54)	0.005
History of heart failure	20 (6%)	6 (11%)	1.76 (0.77, 4.00)	0.18
History of atrial fibrillation	33 (11%)	13 (24%)	2.39 (1.29, 4.43)	0.006
History of stroke	26 (8%)	8 (15%)	1.74 (0.83, 3.63)	0.14
COPD/Asthma	41 (13%)	16 (29%)	2.47 (1.38, 4.41)	0.003
Serum creatinine^a^	0.9 (0.7, 1.1)	0.9 (0.8, 1.0)	0.78 (0.49, 1.25)	0.30
Heart disease				
No heart disease	81 (26%)	5 (9%)	Reference	
Ischemic heart disease	29 (9%)	7 (13%)	3.96 (1.26, 12.51)	0.02
Severe aortic stenosis	18 (6%)	5 (9%)	5.98 (1.71, 20.95)	0.005
Hypertensive heart disease	79 (26%)	24 (44%)	4.63 (1.76, 12.14)	0.002
Valvular heart disease	51 (17%)	8 (15%)	2.87 (0.94, 8.80)	0.06
Amyloidosis	2 (0.6%)	2 (4%)	19.51 (3.72, 102.27)	<0.001
Others	19 (6%)	2 (4%)	2.12 (0.41, 10.98)	0.37
NYHA class				
1	216 (70%)	26 (47%)	Reference	
2	79 (26%)	24 (43%)	2.12 (1.22, 3.71)	0.008
3–4	9 (3%)	5 (9%)	3.56 (1.37, 9.27)	0.009
Standard echocardiographic parameters				
LVEF Simpson (%)	65 ± 7	63 ± 7	0.95 (0.91, 0.99)	0.02
LV Global longitudinal strain (%)	−21 ± 3	−20 ± 2	1.17 (1.05, 1.30)	<0.001
E/A ratio^a^	0.82 (0.75, 1.2)	0.89 (0.70, 1.1)	1.49 (0.88, 2.55)	0.14
e' average (cm/s)	8 ± 4	6 ± 2	0.73 (0.63, 0.84)	<0.001
LAVi (ml/m^2^)	37 ± 13	48 ± 12	1.05 (1.03, 1.07)	<0.001
E/e' average	12 ± 6	14 ± 5	1.05 (1.02, 1.09)	0.0002
TR velocity (cm/s)	255 ± 37	281 ± 43	1.02 (1.01, 1.02)	<0.001
LV mass (g/m^2^)	113 ± 30	132 ± 41	1.02 (1.01, 1.02)	<0.001
RWT	0.43 ± 0.1	0.45 ± 0.1	4.14 (0.34, 50.69)	0.27

Demographics, clinical and standard echocardiographic parameters.

Data are shown as frequency (column percentage) and as mean ± standard deviation; HR (95% CI): Hazard ratio and its corresponding 95% confidence interval.

COPD, chronic obstructive pulmonary disease; NYHA, New York Heart Association; LVEF, left ventricular ejection fraction; LV, left ventricular; LAVi, left atrial volume index; TR, tricuspid regurgitation; RWT, relative wall thickness.

^a^ Results displayed as median (*P*_25_ − P_75_).

**Table 3:** Univariate survival analysis for the presence of the combined event (heart failure, atrial fibrillation or ischemic stroke). Diastolic function and left atrial strain (LARS).

Characteristic	No event (*n* = 309)	Event (*n* = 55)	HR (95% CI)	*P* value
Diastolic function				
NDF	87 (28%)	1 (2%)	Reference	
DD1	84 (27%)	2 (4%)	2.27 (0.21, 25.03)	0.50
IDT	63 (21%)	13 (24%)	17.37 (2.27, 132.84)	<0.001
DD-EFP	75 (24%)	39 (70%)	37.64 (5.17, 274.07)	<0.001
Diastolic function combined with LARS				
NDF	87 (28%)	1 (2%)	Reference	
DD1	84 (27%)	2 (4%)	0.44 (0.04, 4.86)	0.50
IDT with LARS > 24%	56 (18%)	7 (13%)	4.71 (0.98, 22.66)	<0.001
IDT with LARS ≤ 24%	7 (2%)	6 (11%)	28.49 (5.74, 141.38)	0.05
DD-EFP	75 (24%)	39 (70%)	16.60 (4.00, 68.75)	<0.001
Left atrial reservoir strain (LARS)				
LARS (%)	32 ± 10	23 ± 9	0.91 (0.88, 0.94)	<0.001
LARS (%)				
>24	247 (80%)	23 (42%)	Reference	
18–24	40 (13%)	15 (27%)	3.63 (1.89, 6.96)	<0.001
≤18	22 (7%)	17 (31%)	7.03 (3.74, 13.23)	<0.001
LARS (%)				
>24	247 (80%)	23 (42%)	Reference	
≤24	62 (20%)	32 (58%)	4.88 (2.85, 8.35)	<0.001

Data are shown as frequency (row percentage) and as mean ± standard deviation; HR (95% CI), hazard ratio and its corresponding 95% confidence interval. NDF: normal diastolic function; IDT: indeterminate diastolic function and diastolic dysfunction with indeterminate filling pressure; DD1: grade 1 diastolic dysfunction; DD-EFP: diastolic dysfunction with elevated filling pressure; LARS: left atrial reservoir strain.

### 3.4 Diastolic function and combined event

**Figure 1:**
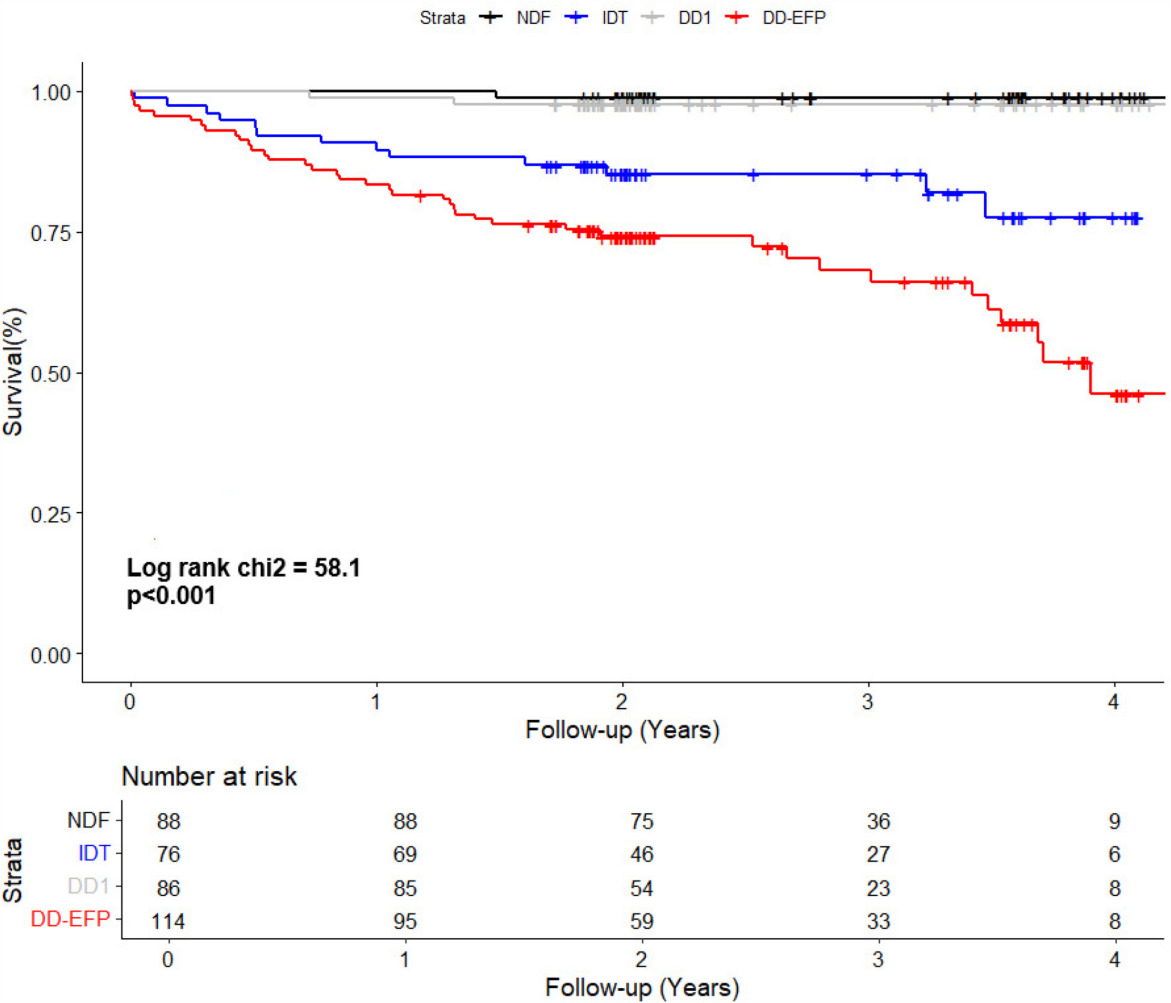
Kaplan–Meier curve shows the survival of patients according to their diastolic function status. NDF, normal diastolic function; IDT, indeterminate diastolic function and diastolic dysfunction with indeterminate filling pressure; DD1, grade 1 diastolic dysfunction; DD-EFP, diastolic dysfunction with elevated filling pressure.

Patients with NDF and DD1 experienced only a few events during follow-up (1/88 and 2/86, respectively). Conversely, patients with DD-EFP and IDT had a high incidence of the combined event (34% and 17% respectively, Table 3). The Kaplan–Meier curve shows the survival from the combined event according to their diastolic function status (Figure 1). Using NDF as the reference group, DD1 patients presented no significant higher risk (*P* = 0.50). Both IDT and DD-EFP patients showed an increased risk of events in the univariate analysis (both *P* < 0.001, Table 3).

In multivariate analysis Model 1, when LARS is not taken into account, individuals with DD1 presented no significant higher risk compared to the reference group (NDF). Both the IDT and the DD-EFP groups exhibited a significant increased risk, HR 9.32 (95% CI, 1.13–76.98; *P* = 0.04) and HR 12.73 (95% CI, 1.50–108.17; *P* = 0.02) respectively (Table 4). Importantly, these results were observed after adjusting for specific heart disease (only amyloidosis remained associated with the combined event). In addition, history of AF, HF or ischemic stroke were not significantly associated with the combined event. Diastolic function inter-rater agreement (Kappa coefficient): 0.68 (CI 95%, 0.48, 0.87). Elevated filling pressure inter-rater agreement (Kappa coefficient): 0.80 (0.68, 0.98).

**Table 4:** Cox regression multivariate models for the prediction of the combined event (heart failure, atrial fibrillation or ischemic stroke) (*n* = 364).

Characteristic	Model 1^a^		Model 2^a^		Model 3^a^	
	HR (95% CI)	*P*-value	HR (95% CI)	*P*-value	HR (95% CI)	*P*-value
Diastolic function						
NDF	Reference		–	–	Reference	
DD1	0.50 (0.03, 8.96)	0.64	–	–	1.88 (0.11, 33.33)	0.67
IDT	9.32 (1.13, 76.98)	0.04	–	–	17.94 (2.33, 138.33)	0.006
DD-EFP	13.89 (1.62, 119.10)	0.02	–	–	18.25 (2.24, 148.85)	0.007
Diastolic function combined with LARS						
NDF	–	–	Reference			
DD1	–	–	0.66 (0.04, 12.18)	0.78		
IDT with LARS > 24%	–	–	5.84 (0.65, 52.26)	0.11		
IDT with LARS ≤ 24%	–	–	58.35 (5.89, 578.18)	<0.001		
DD-EFP	–	–	19.42 (2.18, 172.69)	0.001		
LARS						
>24%					Reference	–
≤24%					2.63 (1.21, 5.72)	0.02
C-index (95% CI)	0.87 (0.82, 0.92)		0.88 (0.83, 0.92)		0.88 (0.83, 0.93)	

HR (95% CI), hazard ratio and its corresponding 95% confidence interval; NDF, normal diastolic function; IDT, indeterminate diastolic function and diastolic dysfunction with indeterminate filling pressure; DD1, grade 1 diastolic dysfunction; DD-EFP, diastolic dysfunction with elevated filling pressure; LARS, left atrial reservoir strain.

^a^ Models adjusted by age, gender, body mass index, hyperlipidemia, chronic obstructive pulmonary disease, heart disease, New York Heart Association class, left ventricular ejection fraction. Detailed models can be found in the Supplementary Tables S5, S6.

### 3.5 LARS and combined event

**Figure 2:**
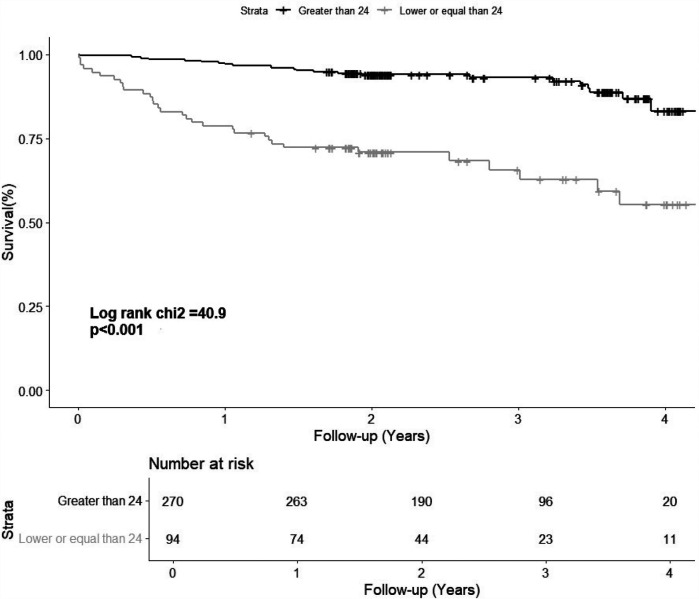
The Kaplan–Meier curve shows the survival of patients according to their dichotomized left atrial reservoir strain value (>24% and ≤24%). LARS, left atrial reservoir strain.

Patients who experienced events had lower LARS values (23% ± 9% vs. 32% ± 10%, *P* < 0.001). After categorising LARS into three groups, patients with a LARS > 24% had the lowest event rate (9%), followed by those with a LARS 18%–24% (27%) and those with a LARS ≤ 18% (44%) (Table 3). Univariate analysis showed that patients with LARS 18%–24% had a significantly higher risk than those with a LARS > 24% [HR 3.63 (95% CI, 1.89–6.96; *P* < 0.001), Table 3]. The Kaplan–Meier curve showed the survival from the combined event according to their dichotomized LARS value with a cutoff value of 24% (Figure 2). The HR of patients with LARS < 24% was 4.88 (95% CI, 2.85–8.35; *P* < 0.001, Table 3).

### 3.6 LARS to improve risk assessment

**Figure 3:**
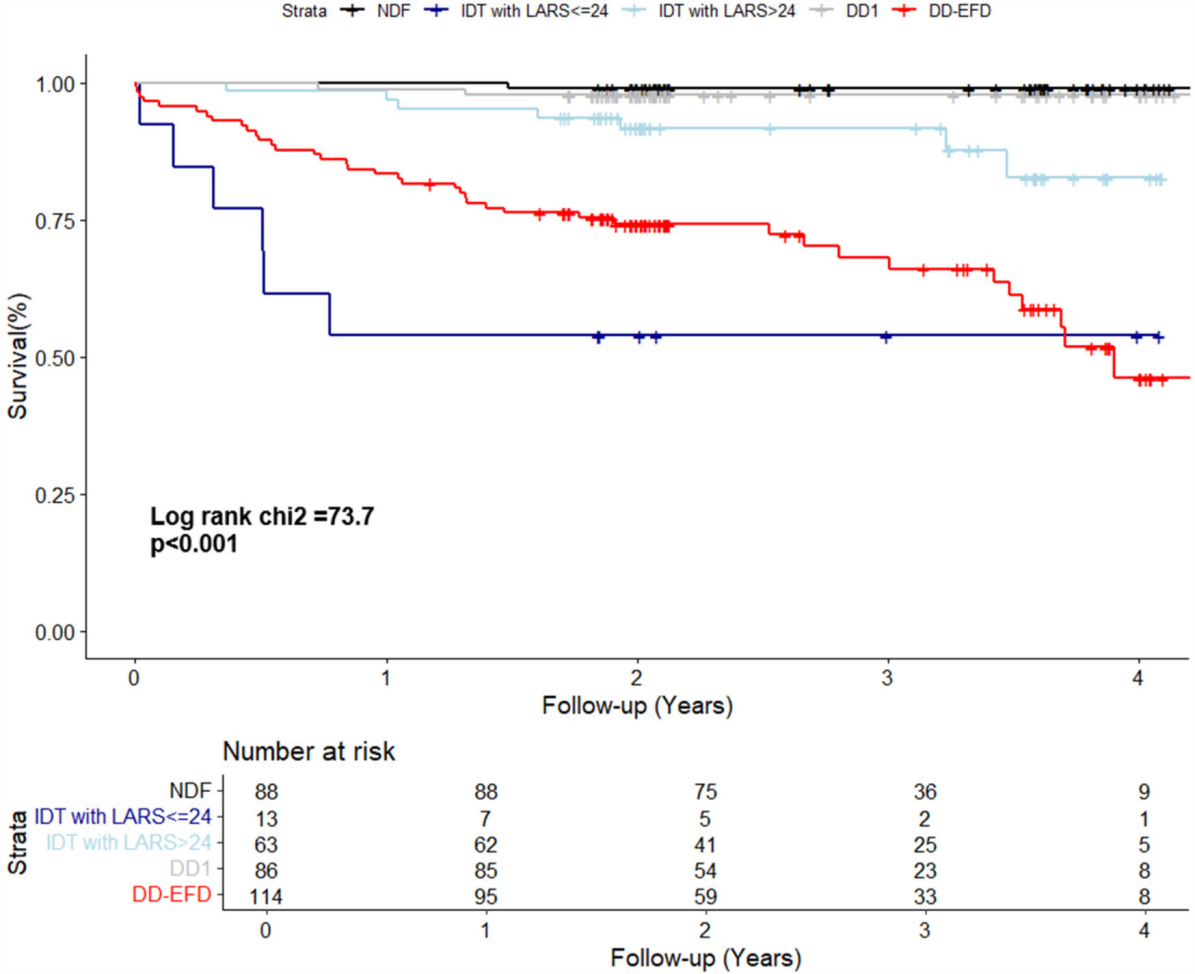
The kaplan–meier curve displays the survival of patients according to their diastolic-LARS combined status. NDF, normal diastolic function; IDT, indeterminate diastolic function and diastolic dysfunction with indeterminate filling pressure; DD1, grade 1 diastolic dysfunction; DD-EFP, diastolic dysfunction with elevated filling pressure; LARS, left atrial reservoir strain.

IDT patients are a heterogeneous group, including individuals with normal and elevated filling pressures, and therefore with different risk profiles. To improve their risk stratification, their prognosis was assessed by adjusting for dichotomized LARS value, with a cutoff point of 24%. The Kaplan–Meier curve displays the survival of patients according to their diastolic-LARS combined status showing that IDT patients with LARS < 24% and DD-EFP patients were those with worse prognosis (Figure 3; Table 3). Model 2 of multivariate analysis showed that diastolic function interacted with LARS is an independent and strong predictor of combined events, allowing better risk stratification of patients in the IDT group (Table 4). In Model 3 of the multivariate analysis, LARS < 24% had an added and independent prognostic value, significantly improving the accuracy of event prediction, HR 2.63 (C95% CI, 1.21–5.72, *P* = 0.02). (Table 4). Model 2 exhibits a better fit with respect to Model 1 and Model 3 (*P* < 0.001). The net reclassification index also showed that Model 2 (the one with the interaction term) improves the classification of individuals into clinically relevant risk categories compared to an existing model (Model 1 and Model 3) (Table 5).

**Table 5:** Net reclassification index (NRI) values of each assessed multivariate models.

NRI		
Model 1	Model 2	Model 3
*Reference*	0.46 (0.07, 0.71)	0.12 (0.05, 0.85)
	0.33 (0.04, 0.45)	*Reference*

Model 1: diastolic function adjusted by other confusor variables: age, gender, body mass index, hyperlipidemia, COPD, type of heart disease, NYHA class, LVEF.

Model 2: interaction of the LARS variable with diastolic function adjusted by the same confusor variables.

Model 3: nested model from the Model 1, with the addition of the LARS variable categories.

LARS intraclass-correlation coefficient (ICC) was 0.97 (95% CI, 0.94–0.99).

### 3.7 Secondary endpoint: all-cause mortality

During the follow-up period, 36 patients died (9.8% of the sample). In the univariate analysis, DD-EFP almost reached a significant association with all-cause mortality (*P* = 0.052). LARS, age, amyloidosis, NYHA class 3–4, LVEF and some diastolic parameters were significantly associated with mortality in the univariate analysis (Supplementary Tables S5, S6). However, in the multivariate analysis, only hyperlipidemia, amyloidosis and NYHA class 3–4 were independently associated with all-cause mortality (Supplementary Table S7).

## 4 Discussion

The majority of studies that have evaluated left atrial reservoir strain (LARS) in the context of diastolic function assessment have been conducted with the objective of improving the determination of filling pressures. When LARS has been employed to assess its prognostic value, it has typically been applied to specific populations and in the context of specific heart diseases, such as heart failure with preserved ejection fraction (HFpEF) or aortic stenosis.

In this study we evaluated the prognostic ability of diastolic function to predict a combined event of heart failure, atrial fibrillation, or ischemic stroke and the added value of left atrial reservoir strain (LARS) for risk stratification in patients with preserved left ventricular ejection fraction (LVEF) and sinus rhythm, in a general cardiologic population. The main findings are as follows: (1) diastolic function is independently associated with the combined event, with patients in the DD-EFP group being at highest risk; (2) LARS improves the risk stratification of patients in the IDT group, with patients with LARS < 24% being those at highest risk. Diastolic function interacted with LARS is strongly associated with the combined event, allowing better risk stratification of patients. Interestingly, these results were consistent regardless of the patient's heart disease (excepting amyloidosis); (3) event rate is high in patients with DD-EFP and IDT patients with LARS < 24%; (4) Diastolic function and LARS were not independently associated with mortality after adjusting for covariates (including NYHA class, age, type of heart disease, LVEF).

### 4.1 Diastolic function and risk of combined event

The prognostic assessment of diastolic function using the 2016 ASE/EACVI guidelines (11) in a general population is limited, as most studies have either focused on examining a specific heart disease or used previous diastolic function guidelines, often addressing HF, AF, and ischemic stroke separately (2). Our study investigated patients referred for echocardiography regardless of a specific heart disease. Notably, patients with DD1 only experienced a very few events, without a significant increase in risk compared with the NDF group. This observation could be influenced by the sample size and the relatively low event rate within this group. However, this finding is consistent with the result of a previous study (12), which demonstrated that only patients with moderate or severe diastolic dysfunction had a worse prognosis, while those with mild diastolic dysfunction did not. Patients with DD1 have normal filling pressure, which could imply a lower risk of left atrial dilatation, HF, AF, and ischemic stroke. Our study found that patients with elevated filling pressure bore the highest risk of events, with a high event rate at 2.4 years (34%). These results are relevant because they show that these patients are at high risk of events and are therefore a population that could probably benefit from preventive measures. Furthermore, in our study, patients with IDT also exhibited an elevated risk in the multivariate analysis (Model 1). However, patients in the IDT group are heterogeneous, including individuals with normal and elevated filling pressures, and therefore with different risk profiles. There is therefore a need for improvement in the risk stratification of these patients.

### 4.2 LARS and risk of combined event

The E-wave occurs during the passive filling phase of the left ventricle and reflects the LA-VI gradient during protodiastole. Its main determinants are LV relaxation and LA pressure. It participates in key diastolic parameters such as E/e’ ratio and E/A ratio, which are fundamental criteria in the assessment of LV diastolic function. As main limitations, both E velocity and E/e’ ratio have low correlation with LV filling pressures when LVEF is preserved. Unlike the E wave, the LARS occurs in the left ventricular systole phase and reflects the deformation of the left atrial myocardium during its filling in the reservoir phase. It is not directly related to LV diastole, stiffness or relaxation. Instead, it is influenced by left ventricular systolic parameters and by the structure and function of the left atrium. Its main determinants are in fact the LV GLS and LA stiffness and relaxation (13). Although the relationship of LV filling pressures to LARS is an indirect relationship, attempts have been made to use LARS for its noninvasive estimation, with a cut-off value <18% to identify elevated left atrial pressure (5). However, invasive studies have shown that in contrast to patients with HFrEF, the correlation with filling pressures in patients with HFpEF is low, being even non-existent from LV GLS values >18% (6). Despite this, a cut-off value for LARS of 18% is still useful in some patients, although most patients with HFpEF have higher LARS values (13). In spite of these limitations as a diastolic parameter, LARS has been shown to provide independent and fundamental information of atrial cardiomyopathy, beyond LA dilatation, with great prognostic value in different related pathologies, such as heart failure, atrial fibrillation and embolic stroke. Moreover, it has also been shown to be an indirect reflection of the degree of left atrial fibrosis (14), which makes it a very useful parameter, given the prognostic role of atrial fibrosis in different heart diseases. We have used this prognostic ability of LARS to improve risk stratification of a combined HF/AF/ischemic stroke event in patients with IDT, confirming that LARS helps to identify those at highest risk within this heterogeneous but large group.

#### 4.2.1 Cutoff value

Despite its limitations, LARS serves as a valuable parameter for assessing diastolic dysfunction and estimating left atrial pressure. The EACVI consensus document advocates for LARS, utilising a cutoff value of <18%, to identify patients with elevated pressure when standard parameters (left atrial volume index, E/e’ ratio, and tricuspid regurgitation velocity) are not available. However, this cutoff value is less accurate when used in the assessment of patients with preserved LVEF and most patients with HFpEF have LARS values higher than 18% (13). The British Society of Echocardiography's recent diastolic function assessment guidelines (15) for normal left ventricular ejection fraction (LVEF) maintained the specific cut-off value of LARS < 18% for high filling pressures and introduced a cut-off value of LARS > 30% or left atrial pump strain >14% for normal filling pressures. This acknowledges the significant overlap in LARS values between 19% and 29%. In the present study, LARS is not employed for the estimation of filling pressures. Rather, it is employed to estimate the event risk in patients with preserved LVEF, regardless of the cardiac structural abnormalities they present, and accounting for relevant comorbidities. We found that those with LARS < 18% were at the highest risk of suffering events. However, patients with LARS 18%–24% also exhibited a significantly elevated risk (Table 3). Further analysis of 18% vs. 24% cutoff values showed that a LARS value of ≤24% provided optimal and more balanced results: Sensitivity 0.31 (0.19–0.43) vs. 0.58 (0.45–0.71), Specificity 0.93 (0.9–0.96) vs. 0.80 (0.75–0.84), positive predictive value 0.44 (0.28–0.59) vs. 0.34 (0.24–0.44), negative predictive value 0.88 (0.85–0.92) vs. 0.92 (0.88–0.95), accuracy 0.84 (0.80–0.87) vs. 0.77 (0.72–0.81) (Supplementary Table S8). The lower limit of normality for LARS is controversial in the scientific literature. Two multicentric studies of healthy individuals, Morris et al. (16) and Sugimoto et al. (EACVI NORRE study) (17) reported 23.1% and 22.7% (in ≥60 years) respectively as the lower limit of LARS. However, recently another multicenter study (18) has reported a LARS value of 17% as the lower limit of normal but warn that the reference ranges are very wide, despite good reproducibility. From a clinical perspective, LARS > 24% is associated with normal filling pressure in patients with preserved LVEF (6, 19) and with incident heart failure in patients with asymptomatic diastolic dysfunction (20). After reviewing the literature and analysing our data, we decided to use 24% as the cutoff point so that more patients could benefit from potential preventive measures.

#### 4.2.2 Improving risk stratification

The IDT group is heterogeneous and includes patients with both normal and elevated filling pressure. In Model 1, when LARS is not considered, patients with IDT were at higher risk than NDF patients. However, in Model 2, when refining IDT patients according to dichotomized LARS, only IDT patients with LARS < 24% had a high risk beside DD-EFP patients (Figure 3; Table 4). Model 2 exhibits a better fit with respect to Model 1 and Model 3 (likelihood ratio test p < 0.001). The net reclassification index was also assessed and showed that Model 2 (with the interaction term) improves the classification of individuals into clinically relevant risk categories, compared to Models 1 and 3 (Table 5). In addition to the improved performance of model 2, LARS is only used in patients with IDT, which is an important practical aspect as it avoids time-consuming proceedings. IDT patients represent a large population (21% of patients with preserved LVEF referred for ETT) (2), thus identifying those at highest risk is a major issue.

### 4.3 Cardiac diseases and prognosis

We tried to take into account the influence of different types of heart disease in the analysis, rather than focusing on a single type of heart disease. Amyloidosis has been studied separately due to its high risk of heart failure (HF) and atrial fibrillation (AF), as including it in another category could overestimate the risk of other heart diseases. The same applies to severe aortic stenosis, which is why it is studied in a different category to the other valve diseases (which excludes patients with moderate-to-severe or severe mitral regurgitation, mitral stenosis or mitral valve prosthesis). The multivariate analysis showed that both diastolic function and LARS predicted the combined event independently of the type of heart disease (except for amyloidosis), suggesting that the results could be generalised.

### 4.4 Role of comorbidities in the prognosis of patients with preserved LVEF

Comorbidities play an important role in the evolution of patients with preserved LVEF, especially if they have underlying heart disease (e.g., stage B heart failure). We have made an effort to adjust for those that we consider most relevant: renal function (serum creatinine levels), COPD, obesity (body mass index), diabetes, hypertension, and hyperlipidemia. In our work, only hyperlipidemia and COPD were independently associated with the combined event. COPD is a recognized comorbidity that is well-known for its association with an increased risk of developing atrial fibrillation (AF) or heart failure (21).

### 4.5 Secondary endpoint: all-cause mortality

Although LARS was associated with all-cause mortality in univariate analysis and DD-EFP almost reached significant association, only hyperlipidemia, amyloidosis and NYHA class 3–4 were independently associated with mortality in multivariate analysis (Supplementary Tables S5–S7). This may be explained by the short follow-up period for the assessment of this endpoint. In addition, mortality in patients with preserved LVEF is often related to non-cardiac conditions.

### 4.6 Clinical implications

Our work has a strong practical approach and highlights the prognostic importance of diastolic function and LARS in patients with sinus rythm and preserved LVEF. One of the strengths of the study is that it is not limited to a population with a specific pathology (ischemic heart disease, HFpEF, aortic stenosis..) but rather the population is a general cardiological population referred for echocardiography. The results take into account the different heart diseases and are therefore more generalizable.

**Figure 4:**
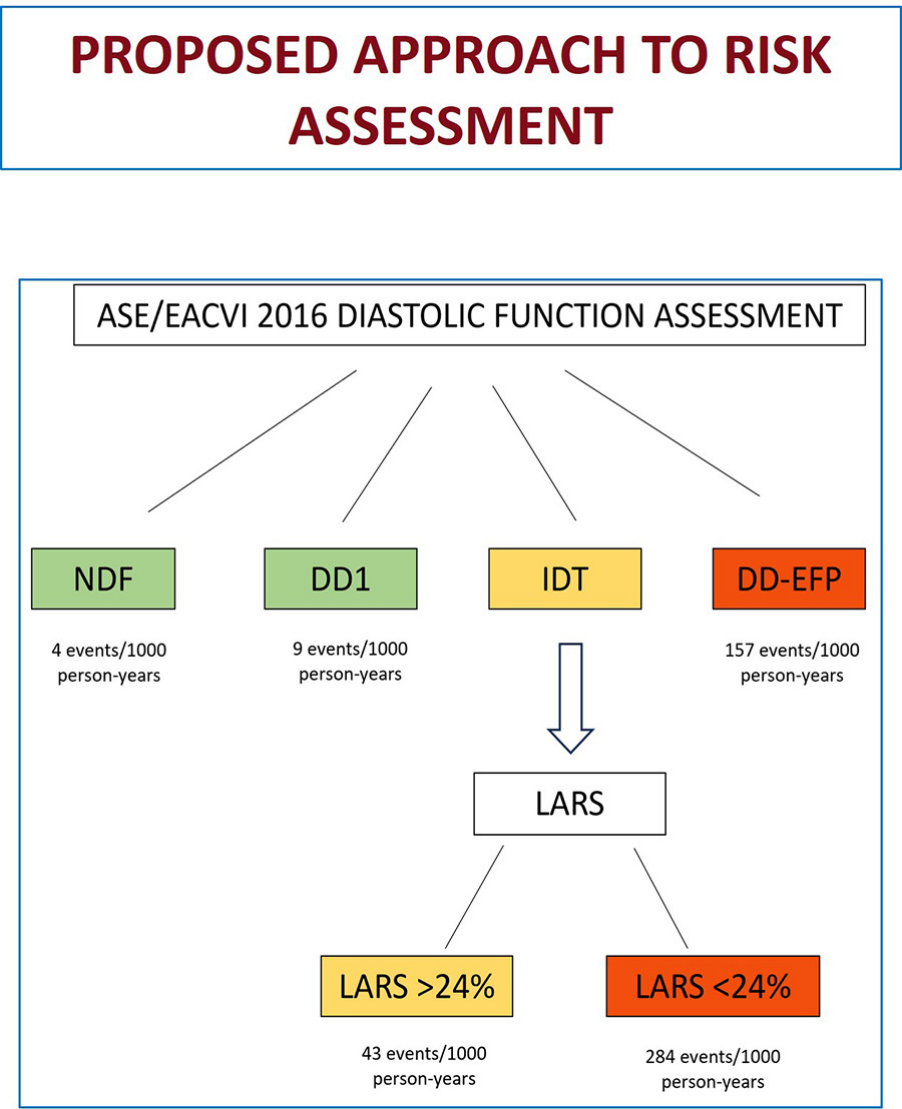
Proposed approach for risk assessment (combined event of HF/AF/ischemic stroke) when evaluating diastolic function in patients with normal LVEF. HF, heart failure; AF, atrial fibrillation; LVEF, left ventricular ejection fraction; NDF, normal diastolic function; DD1, grade 1 diastolic dysfunction; IDT, indeterminate diastolic function/filling pressure; DD-EFP, diastolic dysfunction with elevated filling pressure; LARS, left atrial reservoir strain.

We propose the following approach to stratify the risk of a combined event in patients with preserved LVEF: patients with DD-EFP have a high probability of suffering the combined event and would not benefit from further risk assessment using LARS. No further analysis would be required in patients with NDF and DD1, who have a very low probability of events. However, in patients with IDT, we propose to use LARS < 24% for a better stratification (Figure 4). High-risk patients (DD-EFP and IDT with LARS < 24%) could benefit from different preventive measures. First, prolonged electrocardiographic monitoring to detect silent AF could reduce the risk of stroke related to silent AF and the risk of heart failure related to silent AF with rapid ventricular rate. Second, as our findings show in agreement with current HF guidelines, comorbidities are strongly associated with the occurrence of the combined event, suggesting that a good control is essential. Finally, the use of new drugs such as SGLT2 inhibitors and GLP-1 receptors agonists in patients with stage B heart failure could be an attractive option, but more studies are needed to confirm it.

### 4.7 Limitations

This single-centre, retrospective study has some limitations to consider.

Our study did not assess filling pressures invasively, and their ultrasound estimation has limitations (5, 22, 23). However, this non-invasive estimation is supported by diastolic function guidelines, and the aim of the present study is to assess the value of their estimation and their association with clinical events. Silent AF was sought at the discretion of the responsible physician; In the absence of a specific protocol for searching for silent AF, we cannot rule out the presence of detection bias. Some patients had a history of HF, AF or ischemic stroke, which could increase the risk of new events. However, diastolic function and LARS were independently associated with the combined endpoint, after adjusting for prior AF, stroke, and HF. During the study period (2018–2020), there was no indication for disease-modifying treatment in patients with HFpEF, such as ISGLT2, GLP-1 receptor agonists or finerenone. For this reason, no data were collected on the treatment of patients. Finally, the results of LARS analysis may be vendor-dependent and not fully generalized to other software.

## 5 Conclusion

In a general cardiology population of patients with preserved LVEF and sinus rhythm, diastolic function is strongly and independently associated with a composite event of HF, AF, and ischemic stroke. Patients classified as DD-EFP are at highest risk, with high event rates during follow-up. Inclusion of LARS improves risk stratification, particularly in patients with IDT, where a cutoff point of ≤24% effectively identifies a high-risk population. Patients with DD-EFP or IDT with LARS ≤ 24% may benefit from intensive management or prolonged ECG monitoring. LARS and diastolic function were not independently associated with all-cause mortality. Future studies are needed to further confirm these findings and validate the proposed approach.

## Data Availability

The raw data will be made available by the authors, upon reasonable request to the corresponding author.
