# Supplementary material for: Diastolic function and cardiovascular events in patients with preserved left ventricular ejection fraction. Improving risk stratification with left atrial strain
Source: Front Cardiovasc Med. 2025 Jul 9;12:1565052. doi: 10.3389/fcvm.2025.1565052 (PMC12283624; doi:10.3389/fcvm.2025.1565052)
Supplement: Supplementary file 1 [file Datasheet1.pdf]

SUPPLEMENTARY MATERIAL

Figure S1. PATIENT SELECTION PROCESS FLOW-CHART.

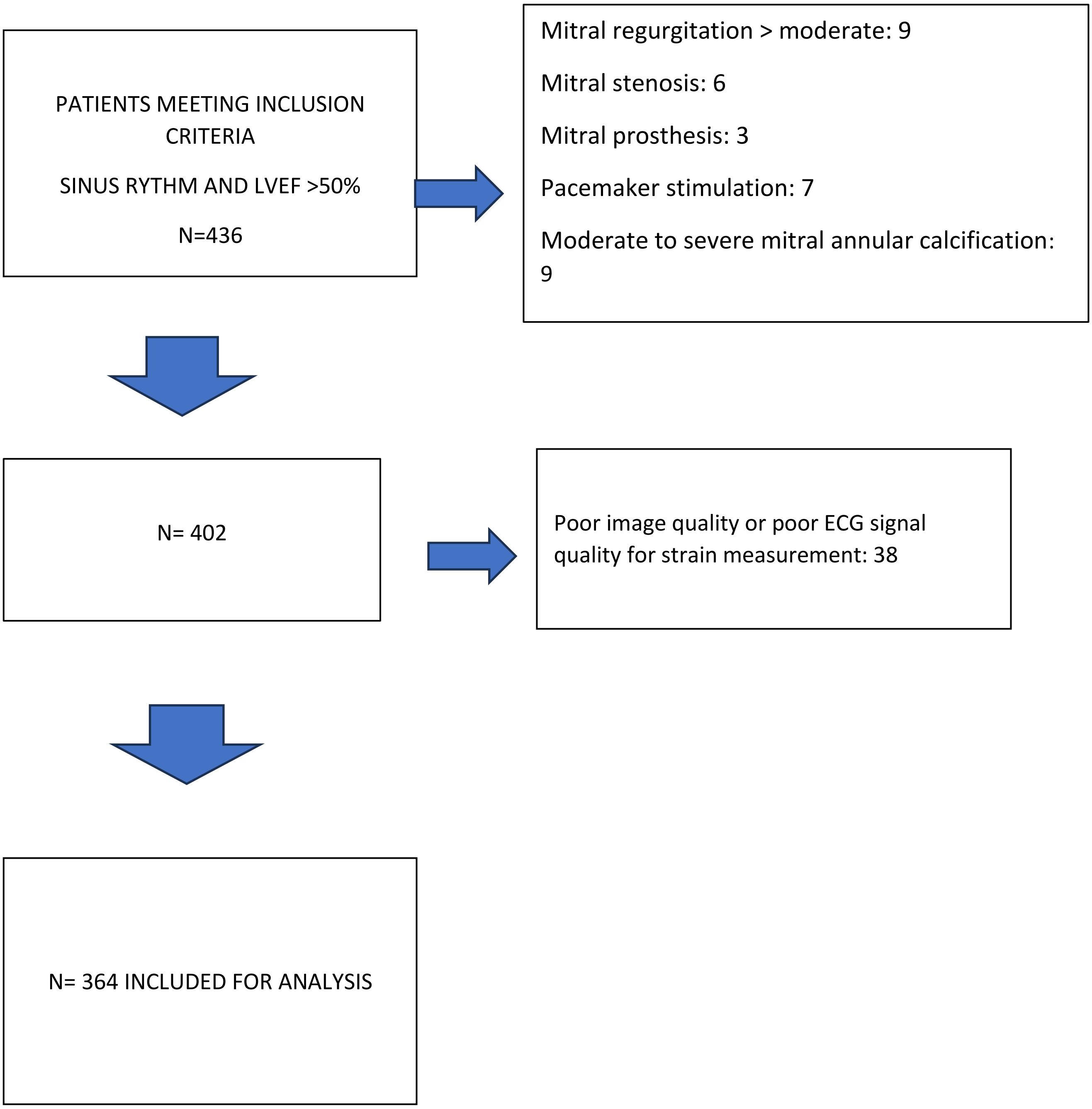

Table S1. Baseline characteristics. Demographics, clinical history and echocardiographic parameters of patients with grade 2 and grade 3 diastolic dysfunction (DD2 and DD3).

Variables	Total (N=114)	DD2 (N=100)	DD3 (N=14)	P Value
**Sex, Female**	79 (69%)	72 (72%)	7 (50%)	0.0946
**Age, y**	76 ±10	77 ±9	70 ±14	0.0450
**Body mass index, (kg/m2)**	29 ±6	29 ±5	30 ±9	0.9209
**Body mass index, (kg/m2)**				0.9762
<25	30 (26%)	26 (26%)	4 (28%)	
25-30	41 (36%)	36 (36%)	5 (36%)	
>=30	43 (38%)	38 (38%)	5 (36%)	
**Hypertension**	93 (82%)	81 (81%)	12 (86%)	0.6700
**Diabetes**	41 (36%)	35 (35%)	6 (43%)	0.5661
**Hyperlipemia**	78 (68%)	67 (67%)	11 (79%)	0.3830
**Heart disease**				0.8495
• *No heart disease*	5 (5%)	5 (6%)	0 (0%)	
• *Amyloidosis*	2 (2%)	2 (2)	0 (0%)	
• *Ischemic heart disease*	10 (10%)	8 (9%)	2 (14%)	
• *Severe aortic stenosis*	14 (14%)	12 (13%)	2 (14%)	
• *Hypertensive heart disease*	50 (48%)	43 (48%)	7 (50%)	
• *Valvular heart disease*	17 (16%)	14 (15%)	3 (21%)	
• *Others*	6 (6%)	6 (7%)	0 (0%)	
**History of heart failure**	19 (17%)	14 (14%)	5 (36%)	0.0412
**History of atrial fibrillation**	23 (20%)	16 (16%)	7 (50%)	0.0030
**History of stroke**	14 (12%)	12 (12%)	2 (14%)	0.6816
**COPD/asthma**	19 (17%)	17 (17%)	2 (14%)	0.7985
**Serum creatinine***	0.9 (0.8, 1.0)	0.9 (0.8, 1)	1.0 (0.8, 1.0)	0.8763
**LVEF (%)**	65 ±7	65 ±7	63 ±6	0.2915
**E/A ratio**	1.2 ±0.9	0.97 ± 0.3	2.7 ±1.9	<.0001
**e´ average**	6.1 ± 4.1	5.7 ±1.6	9.1 ±11	0.1357
**LAVi (ml/m2)**	49 ±12	48 ±11	61 ±15	0.0013
**E/e´average**	17.5 ±7	17± 7	21 ± 8	0.1219
**TR velocity (cm/s)**	290 ±39	288 ±37	306 ± 45	0.1541
**LV mass (g/m2)**	132 ±38	129 ± 35	157 ±49	0.0507
**LARS (%)**	23 ±8	24 ±8	15 ±6	0.0001
**LV global longitudinal strain (%)**	-19.2 ±2.3	-19.2 ± 2.4	-19.2 ± 1.5	0.9427

Data are shown as frequency (column percentage) and mean ± standard deviation. * Results displayed as median (P_{25}-P_{75}). COPD: chronic obstructive pulmonary disease; LVEF: left ventricular ejection fraction; LAVi: left atrial volume index; TR: tricuspid regurgitation; LARS: left atrial reservoir strain; LV: left ventricular.

Table S2. Combined events and specific events in patients with grade 2 and grade 3 diastolic dysfunction (DD2 and DD3).

	Total (N=114)	DD2 (N=100)	DD3 (N=14)	P Value
**Event (Combined event)**	39 (34%)	35 (35%)	4 (29%)	0.7687
**Event (Heart failure)**	17 (15%)	15 (15%)	2 (14%)	0.9440
**Event (Atrial fibrillation)**	21 (18%)	19 (19%)	2 (14%)	0.6700
**Event (Ischemic stroke)**	10 (9%)	10 (10%)	0 (0%)	0.6087

DD2: grade 2 diastolic dysfunction; DD3: grade 3 diastolic dysfunction.

Table S3. Detailed Cox regression multivariate Models 1 and 2 for the prediction of the combined event (atrial fibrillation, heart failure or ischemic stroke).

	Model 1		Model 2	
	**HR (95% CI)**	**P-value**	**HR (95% CI)**	**P-value**
**Clinical history**				
**Age**	1.02 (0.98, 1.05)	0.41	1.01 (0.97, 1.04)	0.72
**Gender: Female vs Male**	0.78 (0.39, 1.58)	0.50	0.73 (0.35, 1.51)	0.39
**Body mass index, (kg/m^2)**				
<25	*Reference*		*Reference*	
25-30	0.99 (0.44, 2.23)	0.97	1.08 (0.47, 2.45)	0.86
>=30	0.77 (0.322, 1.86)	0.56	0.74 (0.30, 1.80)	0.50
**Hyperlipidemia**	3.04 (1.33, 6.97)	0.009	2.93 (1.24,6.91)	0.01
**COPD/Asthma**	1.88 (0.87, 1.07)	0.11	2.37 (1.05, 5.33)	0.04
**Heart disease**				
No heart disease	*Reference*		*Reference*	
Ischemic heart disease	*1.29 (0.31, 5.31)*	*0.73*	*1.02 (0.23, 4.41)*	*0.98*
Severe aortic stenosis	*1.22 (0.25, 6.04)*	*0.80*	*0.89 (0.17, 4.67)*	*0.88*
Hypertensive heart disease	*1.06 (0.30, 3.75)*	*0.93*	*0.72 (0.19, 2.67)*	*0.62*
Valvular heart diseases	*1.36 (0.34, 5.48)*	*0.67*	*0.91 (0.22, 3.85)*	*0.90*
Amyloidosis	*49.98 (5.69, 438.65)*	*<0.001*	*38.38 (4.46, 330.53)*	*<0.001*
Others	*1.39 (0.20, 9.87)*	*0.74*	*1.01 (0.14, 7.44)*	*0.99*
**NYHA**				
**1**	*Reference*		*Reference*	
**2**	*2.29 (1.12, 4.66)*	*0.02*	*2.17 (1.07, 4.43)*	*0.03*
**3-4**	*1.82 (0.54, 6.13)*	*0.34*	*2.00 (0.58, 6.84)*	*0.27*
**Echocardiographic parameters**				
**LVEF SIMPSON* (%)**	0.92 (0.87, 0.96)	<0.001	0.92 (0.87, 0.97)	<0.001
**Diastolic function**				
NDF	*Reference*		-	-
DD1	*0.50 (0.03, 8.96)*	*0.64*	-	-
IDT	*9.32 (1.13, 76.98)*	*0.04*	-	-
DD-EFP	*13.89 (1.62, 119.10)*	*0.02*	-	-
**Diastolic function combined with LARS**				
NDF	-	-	*Reference*	
DD1	-	-	*0.66 (0.04, 12.18)*	*0.78*
IDT with LARS>24	-	-	*5.84 (0.65, 52.26)*	*0.11*
IDT with LARS≤24	-	-	*58.35 (5.89, 578.18)*	*<0.001*
DD-EFP	-	-	*19.42 (2.18, 172.69)*	*0.001*
**C-index (95% CI)**	**0.87 (0.82, 0.92)**		**0.88 (0.83, 0.92)**	

HR (95% CI): Hazard ratio and its corresponding 95% confidence interval; COPD: chronic obstructive pulmonary disease; LVEF: left ventricular ejection fraction; LARS: left atrial reservoir strain; NDF: normal diastolic function; IDT: indeterminate diastolic function and diastolic dysfunction with indeterminate filling pressure; DD1: grade 1 diastolic dysfunction; DD-EFP: diastolic dysfunction with elevated filling pressure.

Table S4. Detailed multivariate Cox regression Model 3 for the prediction of combined event (heart failure, atrial fibrillation or ischemic stroke) with diastolic function and left atrial strain in the additive form.

**Mutivariate Cox regression Model 3**		
	**HR (95% CI)**	**p-value**
**Clinical history**		
**Age**	1.01 (0.97, 1.05)	0.77
**Gender: Female vs Male**	0.68 (0.33, 1.40)	0.30
**IMC**		
<25	Reference	
25-30	1.12 (0.49, 2.59)	0.79
≥30	0.83 (0.34, 2.03)	0.69
**Hyperlipidemia**	3.14 (1.35, 7.36)	0.008
**COPD**	2.04 (0.95, 4.39)	0.07
**Heart disease**		
• *No*	*Reference*	
• *Ischemic heart disease*	*1.18 (0.29,4.85)*	*.0.82*
• *Severe aortic stenosis*	*1.20 (0.24,6.03)*	*0.82*
• *Hipertensive heart disease*	*0.89 (0.25,3.16)*	*0.85*
• *Valve diseases*	*1.19 (0.29,4.88)*	*0.81*
• *Amiloidosis*	*35.06 (4.06, 303.07)*	*0.001*
• *Other heart disease*	*1.44 (0.21,10.20)*	*0.71*
**NYHA**		
**1**	*Reference*	
**2**	*2.47 (1.21, 5.06)*	*0.01*
**3-4**	*1.69 (0.51, 5.65)*	*0.39*
**Echocardiographic parameters**		
**LVEF SIMPSON* (%)**	0.93 (0.88, 0.98)	0.005
**Diastolic function**		
• *NDF*	*Reference*	
• *DD1*	*1.88(0.11, 33.33)*	*0.67*
• *IDT*	*17.94 (2.33, 138.33)*	*0.006*
• *DD-EFP*	*18.25 (2.24, 148.85)*	*0.007*
**LARS**		
• *>24*	*Reference*	-
• *≤24*	*2.63 (1.21, 5.72)*	*0.02*
**C-index (95% CI)***	**0.88 (0.83, 0.93)**	

HR (95% CI): Hazard ratio and its corresponding 95% confidence interval; COPD: chronic obstructive pulmonary disease; NYHA: New York Heart Association; LVEF: left ventricular ejection fraction; LARS: left atrial reservoir strain; NDF: normal diastolic function; IDT: indeterminate diastolic function and diastolic dysfunction with indeterminate filling pressure; DD1: grade 1 diastolic dysfunction; DD-EFP: diastolic dysfunction with elevated filling pressure; LARS: left atrial reservoir strain.

Table S5. Univariate survival analysis for all-cause mortality. Clinical and standard echocardiographic parameters (n = 364).

	Survivors (n = 328)	Dead (n 36)	HR (95% CI)	*P* value
**Demographics**				
**Sex, female, n (%)**	177 (90.8)	18 (9.2)	0.81 (0.42-1.56)	0.53
**Age, y**	67 ±14	74 ±11	1.06 (1.03-1.09)	<0.001
**Body mass index, (kg/m^2)**				
• <25	99 (87)	15 (13)	Reference	
• 25-30	111 (82)	25 (18)	0.69 (0.32-1.49)	0.34
• ≥30	98 (87)	15 (13)	0.67 (0.29-1.51)	0.33
**Clinical history**				
**Hypertension**	216 (90.0)	24 (10.0)	1.01 (0.50-2.02)	0.98
**Diabetes**	80 (87.0)	12 (13.0)	1.45 (0.72-2.9)	0.29
**Hyperlipidemia**	188 (88.3)	25 (11.7)	1.65 (0.81-3.36)	0.16
**Heart disease**				
• *No heart disease*	77 (89.5)	9 (10.5)	Reference	
• *Ischemic heart disease*	35 (97.2)	1 (2.8)	0.25 (0.03-2.04)	0.19
• *Severe aortic stenosis*	21 (91.3)	2 (8.7)	0.99 (0.21-4.63)	0.99
• *Hypertensive heart disease*	92 (89.3)	11 (10.7)	1.02 (0.42-2.46)	0.96
• *Valvular heart disease*	55 (93.2)	4 (6.8)	0.66 (0.20-2.15)	0.49
• *Amyloidosis*	0 (0)	4 (100)	23.2 (6.73-79.8)	<0.001
• *Others*	18 (85.7)	3 (14.3)	1.54 (0.41-5.70)	0.52
**History of heart failure**	21 (80.8)	5 (19.2)	2.23 (0.87-5.74)	0.09
**History of atrial fibrillation**	40 (87.0)	6 (13.0)	1.36 (0.56-3.26)	0.49
**History of stroke**	31 (91.2)	3 (8.8)	0.82 (0.25-2.68)	0.74
**COPD**	51 (89.5)	6 (10.5)	1.11 (0.46-2.67)	0.81
**Serum Creatinine***	0.88 (0.72, 1.05)	0.97 (0.86,1.17)	0.67 (0.35-1.25)	0.20
**NYHA class**				
• 1	225 (93.0)	17 (7.0)	Reference	
• 2	90 (87.4)	13 (12.6)	1.7 (0.83-3.53)	0.14
• 3-4	10 (71.4)	4 (28.6)	4.42 (1.48-13.1)	0.008
**Echocardiographic parameters**				
**LVEF Simpson (%)**	64 ±7	65 ±7	0.95 (0.90-0.99)	0.02
**LV Global longitudinal strain (%)**	-21 ±3	-20 ±2	1.16 (1.05-1.3)	0.05
**E/A ratio**	0.97±0.41	1.08±0.52	0.98 (0.87-1.11)	0.74
**e' average (cm/s)**	8 ±4	7 ±2	0.73 (0.63- 0.84)	<0.001
**LAVi (ml/m^2)**	38 ±13	41 ±14	1.05 (1.03-1.07)	<0.001
**E/e' average**	12 ±6	14 ±6	1.05 (1.02-1.08)	<0.001
**TR velocity (cm/s)**	256 ±39	284 ±34	1.01 (1.01-1.02)	<0.001
**LV mass (g/m^2)**	115 ±31	129 ±44	1.01 (1.01-1.02)	<0.001
**RWT**	0.44 ±0.1	0.45 ±0.1	3.85 (0.31-47.6)	0.29

Data are shown as frequency (row percentage) and as mean ± standard deviation; *results displayed as median (P_{25}-P_{75}). HR (95% CI): Hazard ratio and its corresponding 95% confidence interval. COPD: chronic obstructive pulmonary disease; NYHA: New York Heart Association; LVEF: left ventricular ejection fraction; LV: left ventricular; LAVi: left atrial volume index; TR: tricuspid regurgitation; RWT: relative wall thickness.

Table S6. Univariate survival analysis for all-cause mortality. Diastolic function and LARS. (n = 364).

	Survivors (n = 328)	Dead (n 36)	HR (95% CI)	*P* value
**LARS**				
**LARS (%)**	31 ±10	29 ±14	0.91 (0.88, 0.94)	<0.001
**LARS**				
• *>24*	248 (91.85%)	22 (8.15%)	Reference	
• *18-24*	49 (89.09%)	6 (10.91%)	1.31 (0.53 – 3.23)	0.559
• *≤18*	31 (79.4q%)	8 (20.51%)	2.78 (1.24 – 6.26)	0.013
**LARS**				
• *>24*	247 (91%)	23 (9%)	Reference	
• *≤24*	62 (66%)	32 (34%)	1.88 (0.961-3.67)	0.065
**Diastolic function**				
• NDF	81 (92.05)	7 (7.95)	Reference	
• IDT	70 (92.11)	6 (7.89)	1.01 (0.341-3.02)	0.979
• DD1	83 (96.51)	3 (3.49)	0.452 (0.117-1.75)	0.25
• DD-EFP	94 (82.46)	20 (17.54)	2.34 (0.99-5.54)	0.05
**Diastole combined with LARS**				
• NDF	81 (92.05)	7 (7.95)	Reference	
• DD1	83 (96.51)	3 (3.49)	0.452 (0.117-1.75)	0.25
• IDT with LARS>24	11 (84.62)	2 (15.38)	0.808 (0.236-2.76)	0.733
• IDT with LARS≤24	59 (93.65)	4 (6.35)	2.08 (0.432-10)	0.361
• DD-EFP	94 (82.46)	20 (17.54)	2.34 (0.99-5.54)	0.0526

Data are shown as frequency (row percentage) and as mean ± standard deviation; HR (95% CI): Hazard ratio and its corresponding 95% confidence interval. LARS: left atrial reservoir strain; NDF: normal diastolic function; IDT:indeterminate diastolic function and diastolic dysfunction with indeterminate filling pressure; DD1: grade 1 diastolic dysfunction; DD-EFP: diastolic dysfunction with elevated filling pressure.

Table S7. Cox regression multivariate models for the prediction of all-cause mortality

	Model 1		Model 2 (Diastolic Function interacted with LARS)	
	**HR (95% CI)**	**P-value**	**HR (95% CI)**	**P-value**
**Clinical history**				
**Age**	1.04 (0.99, 1.09)	NS	1.04 (0.99, 1.09)	NS
**Hyperlipidemia**	3.52 (1.23, 10.07)	0.02	3.53 (1.23, 10.13)	0.02
**Heart disease**				
• *No heart disease*	Reference		Reference	
• *Ischemic heart disease*	0.15 (0.01, 1.59)	NS	0.15 (0.01, 1.56)	NS
• *Severe aortic stenosis*	0.59 (0.07, 4.88)	NS	0.56 (0.06, 4.76)	NS
• *Hypertensive heart disease*	0.35 (0.08, 1.55)	NS	0.31 (0.07, 1.49)	NS
• *Valvular heart diseases*	0.44 (0.08, 2.37)	NS	0.42 (0.08, 2.30)	NS
• *Amyloidosis*	95.14 (7.46, 1213.63)	<0.001	91.22 (6.99, 1190.85)	<0.001
• *Others*	1.70 (0.24, 12.20)	NS	1.62 (0.22, 11.95)	NS
**NYHA**				
• 1	Reference		Reference	
• 2	2.39 (0.86, 6.63)	NS	2.36 (0.85, 6.56)	NS
• 3-4	5.60 (1.26, 24.81)	0.02	5.93 (1.32, 26.72)	0.02
**Echocardiographic parameters**				
**LVEF SIMPSON* (%)**	0.93 (0.87, 1.01)	NS	0.93 (0.87, 1.01)	NS
**Diastolic function**				
• *NDF*	Reference		-	-
• *DD1*	0.11 (0.01, 1.03)	NS	-	-
• *IDT*	0.67 (0.15, 3.03)	NS	-	-
• *DD-EFP*	1.08 (0.22, 5.18)	NS	-	-
**Diastolic function interacted w/ LARS**				
• *NDF*	-	-	Reference	
• *DD1*	-	-	0.11 (0.01, 1.09)	NS
• *IDT with LARS>24*	-	-	0.59 (0.12, 2.94)	NS
• *IDT with LARS≤24*	-	-	1.38 (0.12, 15.95)	NS
• *DD-EFP*	-	-	1.14 (0.23, 5.67)	NS
**C-index (95% CI)**	**0.87 (0.79, 0.94)**		**0.87 (0.80, 0.94)**	

HR (95% CI): Hazard ratio and its corresponding 95% confidence interval; NS: non-significant (P-value ≥0.05); COPD: chronic obstructive pulmonary disease; NYHA: New York Heart Association; LVEF: left ventricular ejection fraction; LARS: left atrial reservoir strain; NDF: normal diastolic function; IDT: indeterminate diastolic function and diastolic dysfunction with indeterminate filling pressure; DD1: grade 1 diastolic dysfunction; DD-EFP: diastolic dysfunction with elevated filling pressure.

Table S8. Screening parameters with 95% confidence intervals for the left atrial reservoir strain (LARS) cutoff points.

Cut-off	Sensitivity	Specificity	PPV	NPV	Accuracy
≤18%	0.31 (0.19, 0.43)	0.93 (0.9, 0.96)	0.44 (0.28, 0.59)	0.88 (0.85, 0.92)	0.84 (0.80, 0.87)
≤24%	0.58 (0.45, 0.71)	0.80 (0.75, 0.84)	0.34 (0.24, 0.44)	0.92 (0.88, 0.95)	0.77 (0.72, 0.81)

PPV: positive predictive value; NPV: negative predictive value.
